# Biosensors for Sustainable Food Engineering: Challenges and Perspectives

**DOI:** 10.3390/bios8010023

**Published:** 2018-03-12

**Authors:** Suresh Neethirajan, Vasanth Ragavan, Xuan Weng, Rohit Chand

**Affiliations:** Bionano Laboratory, School of Engineering, University of Guelph, Guelph, ON N1G 2W1, Canada; vkrishna@uoguelph.ca (V.R.); xuanw@uoguelph.ca (X.W.); chandr@uoguelph.ca (R.C.)

**Keywords:** biosensors, food production, sustainability, point of care, food packaging, supply chain, quality assessment, food engineering

## Abstract

Current food production faces tremendous challenges from growing human population, maintaining clean resources and food qualities, and protecting climate and environment. Food sustainability is mostly a cooperative effort resulting in technology development supported by both governments and enterprises. Multiple attempts have been promoted in tackling challenges and enhancing drivers in food production. Biosensors and biosensing technologies with their applications, are being widely applied to tackling top challenges in food production and its sustainability. Consequently, a growing demand in biosensing technologies exists in food sustainability. Microfluidics represents a technological system integrating multiple technologies. Nanomaterials, with its technology in biosensing, is thought to be the most promising tool in dealing with health, energy, and environmental issues closely related to world populations. The demand of point of care (POC) technologies in this area focus on rapid, simple, accurate, portable, and low-cost analytical instruments. This review provides current viewpoints from the literature on biosensing in food production, food processing, safety and security, food packaging and supply chain, food waste processing, food quality assurance, and food engineering. The current understanding of progress, solution, and future challenges, as well as the commercialization of biosensors are summarized.

## 1. Introduction

Food with its production industry is essential to our survival and lives; and its sustainability is significant in continuous human growth on earth. Current food production faces tremendous challenges from growing human population, maintaining clean resources and food qualities, and protecting climate and environment [1]. Some of these challenges come from food production itself; and others derive from other industries related to food production. For example, food recalls cause significant harm to credibility and reputation of food brands, with an average of $15 million per incident in the past few years [2]. Foodborne illnesses cause 48 million sick cases responsible for 3000 fatalities annually.

Food safety is mostly a cooperative effort resulting in technology development from both governments and enterprises. Information technology such as blockchain technology will accelerate communication between food quality, media, and consumers to pose novel challenges in food safety concerns. The top challenges in the sustainability of food production can be summarized as five challenges: production challenge about food safety and security; quality challenge in food diversity and qualities; economic challenge in the governing food system, including its packaging and supply chain; environmental challenge including food waste processing; and engineering challenge in novel food creation and generation [3,4].

Global food production can be driven by several factors. Economic infrastructure, such as information technology, electricity, irrigation, and transportation, is prerequisite in agriculture development [5]. Land tenure systems guarantee land use and determine the economic characteristics of agriculture. Financial services and macroeconomic environments drive food production in its resource mobilization to sustain agribusiness. Furthermore, technical innovations, such as adopting new technology and investing in research and development, drive food production growth and its economic agility.

Multiple attempts have been promoted in tackling challenges and enhancing drivers in food production. For example, nexus studies have discovered the close leakages between food, water, and energy to reveal their relationships and discover solutions [6]. Globally, it has been found that food production accounts for 70% of water abstractions and 90% of water consumption, 8% of water transportation and sewage treatment, and 30% of energy use. Moreover, data mining (DM), or knowledge discovering in data bases (KDD), has been found useful in identifying relationships between food production, food safety, animal welfare, environmental issues, and climate change [7].

This review focuses on biosensors and biosensing technologies with their applications in tackling all five top challenges in food production industry and its sustainability.

## 2. Biosensing Technologies and Food Sustainability

**Figure 1:**
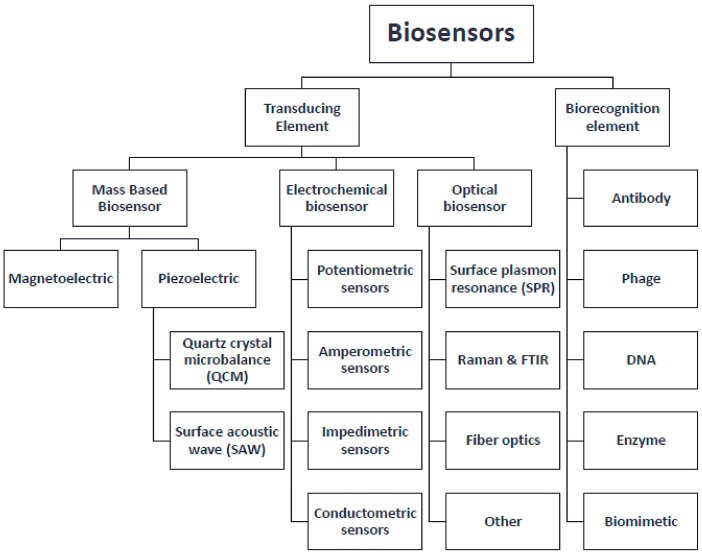
Classification of biosensors based on transducer and bio-recognition elements used in food analysis [10].

A biosensor is basically an analytical device used to quantify molecule of interest (target) in a sample. Generally, it comprises a bio-recognition element (aptamer, antibody, enzyme, etc.) which is specific towards the target. Molecular recognition events between the recognition element and the target compound elicits a physiochemical or biological signal, which is converted into a measurable quantity by the transducer. Signals are displayed in the form of optical (colorimetric, fluorescence, chemiluminescence, and surface plasmon resonance) or electrical (voltammetry, impedance, and capacitance) or any other preferred format (Figure 1). Classification of sensors are discussed elaborately elsewhere [8,9].

**Figure 2:**
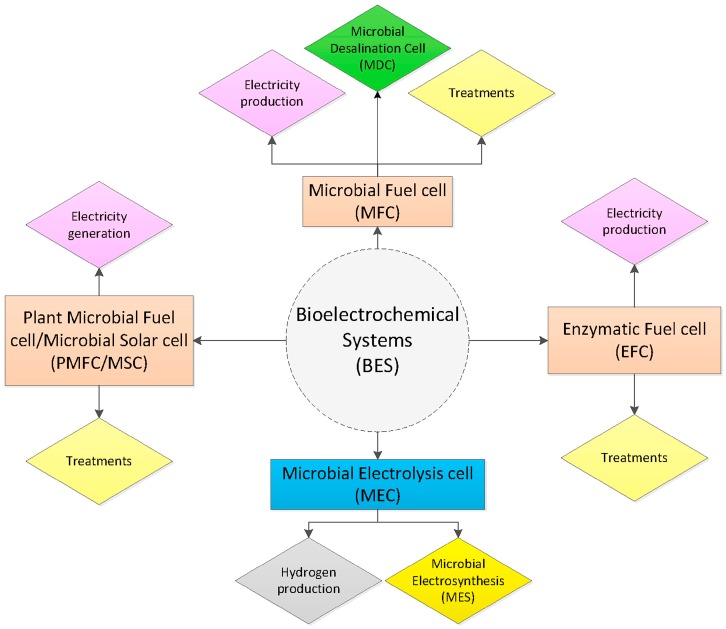
Schematic overview of various types of bio-electrochemical systems (BESs) [11].

A growing demand for biosensing technologies exists in food sustainability, covering all five top challenges, as mentioned above. One of the challenges is new energy sources, as the current reliance on fossil fuels has limited its availability, with potential pollution consequences [11]. To tackle the energy challenge, bioelectrochemical systems (BES) are emerging in the discovery of sustainable electricity sources, chemical production, resource recovery, and waste remediation [12]. These unique systems can convert in both directions between chemical energy and electrical energy using microbes as catalysts derived from organic wastes, such as lignocellulosic biomass and low-strength wastewaters. The systems can be designed to produce electrical energy which can be used to produce hydrogen, caustic and peroxide; to recover metals and nutrients; or to remove recalcitrant compounds. New concepts and innovative designs have been introduced to these systems for novel separators, electrodes, and catalysts (Figure 2).

**Figure 3:**
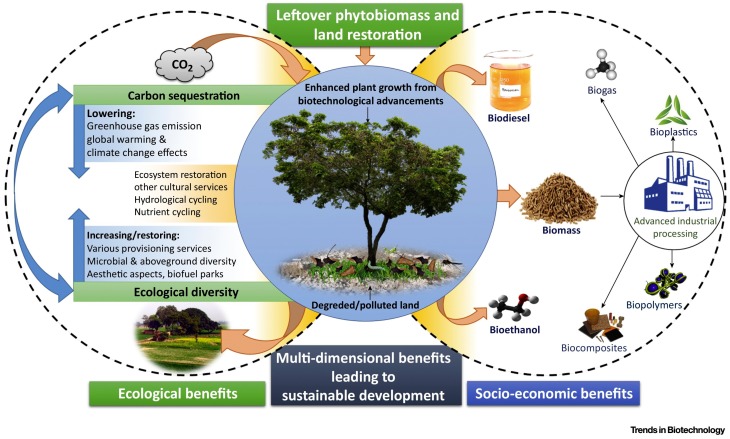
Schematic representation of coupling bioremediation with bioenergy and other value-added products generation for supporting a bio-based economy [14].

Global land degradation is one of the biggest challenges in food production due to rapid urbanization, industrialization, pollution, and unsustainable land use. In the past few decades, land degradation is wide-spread severely to 12.2 billion hectares globally affecting 1.5 billion populations [13]. On the other hand, bioremediation as a promising technology in degraded and polluted land restoration has its potential field limitations. Encouragingly, novel advancements in biotechnology create new directions in sustainable land restoration, such as using enzymes with high specificity, producing microbial consortia, and applying plants with microbial partners [14]. The main concerns are that the land restoration must be contaminant- and site-specific to fit soil and its social conditions of the related areas; and that the restoration activities must be correlated to additional benefits, such as industrial bioproducts, biofuel and biomass products, and soil carbon sequestration (Figure 3).

Biosensors with electrochemical impedance spectroscopy have been widely applied to sustainable food production, which uses a small amplitude AC voltage in its sensing electrode to measure the current response as a frequency [15]. The all-electrical nature of impedance biosensors gives it potential for being developed into portable sensors for environmental monitoring and studies. For example, an impedance biosensor is developed to detect two endocrine disrupting chemicals, BDE-47 and norfluoxetine, with their detection limits of 1.3 and 8.5 ng/mL, respectively [16]. Although impedance biosensors are widely studied in academic research, their commercialization has been limited by several factors: complexity of impedance detection, stability of biomolecule immobilization, smaller analytes, and susceptibility to nonspecific absorption. Current research should focus on overcoming these limitations to facilitate the commercialization of impedance biosensors and their use in sustainable food production.

Inspired by the natural bio-recognition elements, synthetic receptors are designed to mimic their function with better attributes such as sensitivity, robustness, and detection range. While responding to the external stimuli, they have features which switch between on/off status to recognize and transduce the interaction [17]. Novel advances, such as toggle switches, synthetic entities mimicking natural molecules, and gene networks, facilitate the redesign of switchable functions and sensing elements. Examples of related biosensing technologies are: synthetic cell-based biosensors; artificial liposomes; and bioinspired synthetic molecules like biomimetics, molecular imprinting polymers, aptamers, peptide nucleic acids, and ribozymes. These biosensing technologies have been widely applied to molecule sensing, biofuel production, waste degradation, and fine chemical production [17,18].

Foodomics, or the food fingerprint, is about the nutritional values, quality and authenticity, and safety and security of foods [19]. Integrated analytical technologies relying on novel platforms can be used to define food fingerprint in various foods. Associated analytical technologies include advanced analytical techniques, phytochemistry, food chemistry, bioinformatics, and biosensors. Challenges arise when applying the integrated analytical technologies to food productions and its security and sustainability related to globalized environmental changes [20]. Powerful analytical approaches are expected to discover novel biomarkers, to ensure food qualities, to secure food safety, and to facilitate individual peculiarities and personalized prognosis in food production.

### 2.1. Microfluidics in Biosensing Technology

**Figure 4:**
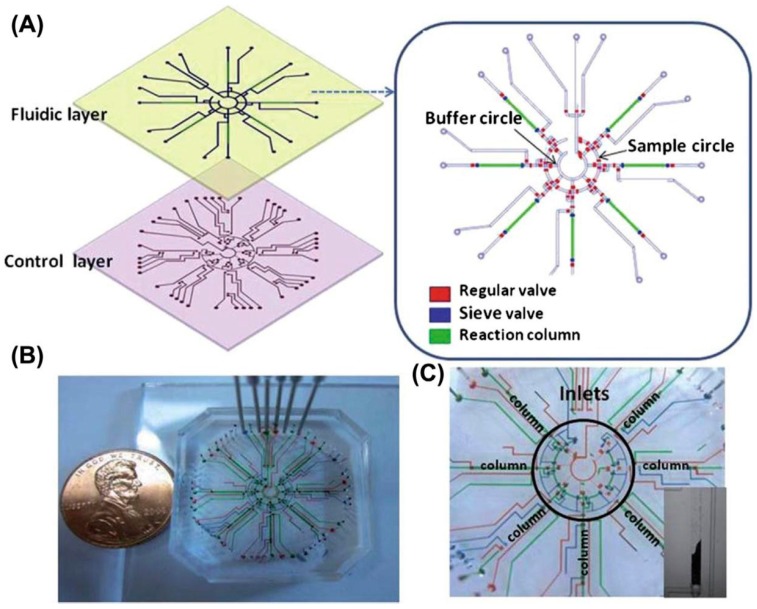
(**A**) Schematic representation of fluidic layers of the immunoreaction chip used in the detection of algal toxins. Valves and columns are clarified by different colors: red (grey in print versions) for regular valves (for isolation), blue (dark grey in print versions) for sieve valves (for trapping protein A beads loaded in the column module) and green (light grey in print versions) indicates the immune columns by loading of microspheres. (**B**) Optical micrograph of the microfluidic chip. The various channels have been loaded with food dyes to help visualize the different components of the microfluidic chip: control line colors are as in (**A**), plus green (light grey in print versions) for fluidic channels. A penny coin (diameter 18.9 mm) is shown for size comparison. (**C**) Optical micrograph of the central area of the chip containing seven immunoreaction columns. Inset: a snapshot of the protein A beads loading process in action [22].

Microfluidics represents a technological system integrating multiple technologies including biosensing, nanotechnology, and microsystems with microscale volumes (10^−9^ to 10^−18^ L) and microsized channels [21]. This integrated system represents the concept of micro total analysis systems (µTAS) in a single platform featured with the integration of multiple miniaturized analytical processes into one single monolithic device (Figure 4). Current challenges in microfluidics development are its microfabrication techniques, electrokinetic and hydrodynamic flows, and electrochemical detection [22]. In its microfabrication techniques, polymers are interesting materials as they are tailorable to fit specific applications. Although its hydrodynamic flows are commonly used because of its higher reproductivity, its electrokinetic feature is distinctive in its roles of controlling multi-channel on a microchip in microfluidics. Furthermore, its electrochemical detection with high compatibility and inherent miniaturization properties surpasses fluorescence to be a natural detection principle, even though the latter has been the most widely used detection technique in microfluidics.

Food safety, as one of the primary goals in food analysis, is a significant health concern in both animal and human lives. The development of analytical technologies in food safety ensures it thrives corresponding to the increasing interest in and focus on safety concerns of food supply. Conventional methods in food safety analysis are labor intensive, time consuming, and requires skilled technicians. The application of microfluidics in food safety analysis sheds new lights on efficient and rapid detection of foodborne toxins, allergens, pathogens, toxic chemicals, heavy metals, and other contaminants [23]. The features of microfluidics, such as its miniaturize-ability, portable and reducible sample and reagent volumes, make it an ideal technology in food sustainability development. Current challenges in the application of microfluidics to food sustainability are complex food matrix preparation and complex fabrication steps. These challenges can be tackled by leveraging physical properties based on specific testing targets, developing diverse microfluidic platforms for real food analysis, and integrating biomolecules such as food proteins and DNA into microfluidic systems [24,25].

The combination of electrochemical microfluidic and cell culture technologies represents a novel analytical technique in food analysis. It can detect food allergen induced changes in cell morphology and cell metabolism to simultaneously facilitate the detection of food safety [26]. For example, two types of cells, ANA-1 macrophages and RBL-2H3 mast cells, are used in cell co-culture for their changes responding to the food allergen dinitrophenylated bovine serum albumin (DNP-BSA). The cell changes can be detected using microfluidic chips fabricated with gold electrodes as a cell-based electrochemical assay without anti-DNP antibodies. The response from the reported assay has options for qualitative and quantitative analysis of food allergens. Results were compared with an enzyme-linked immunosorbent assay (ELISA) detecting inflammatory cytokines released by the cultured cells. The cell allergic responses were confirmed as detectable with real-time and accurate properties, providing a rapid, low cost, and prototyped biosensing microfluidic technology.

### 2.2. Nanomaterials in Biosensing Technology

**Figure 5:**
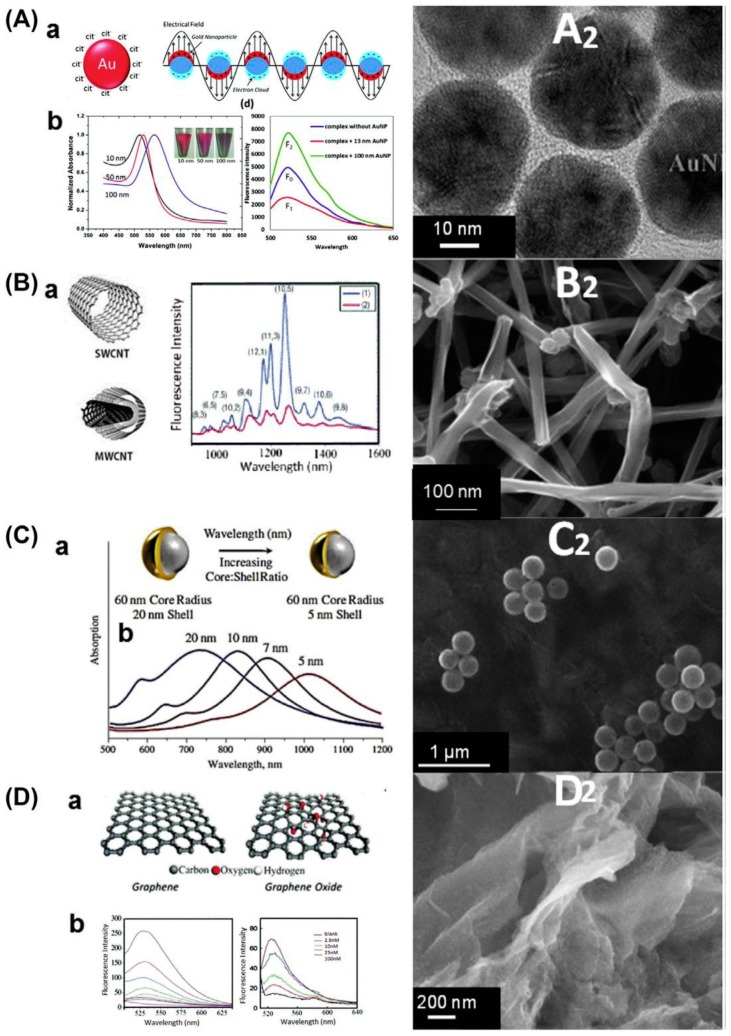
(**A**). Ultra-violet (UV)-Vis absorption spectra corresponding to the gold nanoparticles (AuNPs) of different sizes. (**A2**) Transmission electron microscopy (TEM) images of synthesized AuNPs with average size of 30 nm. (**B**) Illustration of carbon nanotube (CNT) quenching: fluorescence spectra. (**B2**) Scanning electron microscopy (SEM) of multiwall CNTs. (**C**) Optical resonances of gold shell-silica core nanoshells as a function of their core/shell ratio. (**C2**) SEM images of nanoshells. (**D**) Illustration of graphene oxide quenching: fluorescence spectra of graphene and fluorescence spectra of graphene oxide. (**D2**) SEM image of graphene oxide [29].

Nanomaterials, with its technology in biosensing are the most promising tool in dealing with health, energy, and environmental issues related to populations in the world [27]. Nanomaterials are defined as particles less than 100 nm in at least one dimension of size. These nanomaterials are metal-, metal oxide-, and carbon-based polymers with biocomposite properties; and various types of nanoparticles have been developed, such as magnetic iron, aluminum, copper, silica, gold, silver, zinc, zinc oxide, cerium oxide, and titanium dioxide nanoparticles, as well as single/multiple walled carbon nanotubes (CNTs) (Figure 5). Nanotechnology and its development in agriculture has been significantly expanded to various fields [28]. These fields include food production, crop protection, pathogen and toxin detection, water purification, food packaging, wastewater treatment, and environmental remediation. The focus of these agricultural fields is improving the productivity and efficiency of the applications.

One of the emerging application of nanomaterials in biosensing is on analytical chemistry, which plays a quality control role in food analysis [29]. Quality control is significant in food and beverage monitoring because it ensures that product qualities and safety are acceptable for consumers. Chemical analysis can monitor attributes in foods and beverage to guarantee their structure, composition, nutrients, and microbiological characteristics. The introduction of nanomaterials in chemical analysis improves the specificity, sensitivity, and detection limits to achieve femtomolar level detection. Their applications in biosensor technology makes agricultural pathogen detectable in minutes. Nanomaterial-based biosensors are considered as forefront devices with quicker, easier, and less expensive solutions compared to conventional technologies like electrochemical, fluorescence, ultra-violet (UV)-Vis and high performance liquid chromatography (HPLC).

Single and array nanochannels have been applied to improving electrical biosensing in agriculture to detect protein, DNA, pathogens, toxins, and other analytes [30]. Emerging materials such as graphene and its analogues are used to obtain nanochannels and to combine nanochannels with nanoparticles expanding their applications and enhancing their sensitivities. Single nanochannels can be inserted into lipid bilayers to mimic pore forming toxins, which facilitates the extensive research of protein, DNA, pathogens, and toxins. Another application of single nanochannel is drilling nanopores on silicon oxide/nitride membranes using transmission/scanning electron microscopy (TEM/SEM) techniques and electron-beam lithography. The nanopore drilling platforms adopt materials like graphene, boron nitride, molybdenum disulphide, and hafnium oxide, replacing silicon-based materials in the most recent applications. Nanochannel arrays, such as micro- or nano-molding high-ordered mesoporous thin films, metallic substrate anodization, and anodized aluminum oxide nanoporous membranes, are emerging to take advantage of mass production, easy functionalization, and impedimetric, voltammetric, capacitive, conductometric, and resistive measurements.

Chitosan-based nanoparticles are applied to agriculture to improve food productivity with no adverse impact on the environment [31]. Chitosan is ideal as a valuable carrier for controlled delivery slow release of agrochemicals and genetic materials, which is crucial in food production. With its proven qualities in biodegradability, biocompatibility, adsorption abilities, and nontoxicity, chitosan has its advantages in encapsulating agrochemicals and genetic materials, and in protecting active ingredients. It controls the slow release and allows the effective delivery of chemicals and genetic materials in pesticide use and plant transformation. Current challenges in chitosan use are a knowledge gap in its encapsulation of active ingredients in agriculture, scale increase in its production processes, understanding in its toxicology perspectives, and lowering its costs.

Au-based nanomaterials have been known for their unique optical, electrical, and catalytic properties, together with their biocompatibility [32]. These outstanding properties make them excellent candidates in food safety and quality- monitoring through signal reporting and material enhancing. Au-based nanoparticles are widely applied to analytical assays combined with other techniques such as optical imaging, fluorescent detection, plasmonic colorimetric analysis, Electrochemical sensor, surface-enhanced Raman spectroscopy (SERS), and surface plasmon resonance (SPR) technologies. Current challenges in Au-based nanotechnology are how to control precisely the morphology and monodispersed size of the nanoparticles to obtain high qualities signals with vivid color; how to improve the signal intensity of plasmonic colorimetric analysis using Au based nanomaterials in chiral analysis; how to develop multimodal composite nanomaterials with high-sensitive and high-resolution in SERS detection; how to develop multifunctional Au based nanomaterials with Lateral Flow Immunochromatographic Assay (LFICA) labels, signal amplified EC sensor, and colorimetric sensing signals; how to achieve synthesis of high quality Au-based nanochannels with high QY and stability; and how to apply and commercialize Au based nanomaterials and their use in industrial settings.

Inorganic nanomaterials are widely accepted and applied in agricultural food production research with great significance in a diverse range of industrial applications. However, their toxicological impact on human health have not been addressed until very recently [33]. A limited number of nanomaterials currently in use has been documented to be possible nanomaterial candidates in food application; and they are Ag, Al, Au, Co., Cu, Fe, Si, Ti, and Zn, as well as their derivatives. This indicates that most inorganic nanomaterials in use or under development have not been evaluated for their safety, security, and toxicology properties with health concerns. Current focuses of nanomaterials and their use in food production are food condition sensing, food packaging and stability enhancing, and slow release in crop growth boosting. Before toxicology evaluation of these nanomaterials, their applications in food production and sustainability are limited and their health risks remain unknown.

## 3. POC Biosensing Technology for Food Sustainability

Point of care (POC) or point of need (PON) are those technologies that are applicable onsite and available immediately for diagnosis and treatment of individual food production conditions [34]. In food production sustainability, the area that requires POC technologies is food quality control, which is concerned with nutrient monitoring, food safety and security, and food production environment control [35,36]. The demand of POC technologies in this area focuses on rapid, simple, accurate, and portable qualities; the availability of low-cost analytical instruments is currently growing, for which biosensing fully meets demand.

In food safety, for example, rapid POC methods are urgently needed to detect microbiological contaminants [35]. Biosensing technologies are applicable to pathogen detection through detection of bio-active materials in the food industry; these pathogen materials include antigens, antibodies, enzymes, and nucleic acids. These pathogen-related materials can be detected by the recognition unit in biosensors through generating optical, electrical, and thermal signals [37]. The advantages of biosensing are its potency in a shortened timespan of detection and ease of use; but its challenges are its sterilizability, stability, and reusability. These challenges in current application of biosensing in food safety are limited to chemical contaminant detection, but not microbiological contaminant detection.

**Figure 6:**
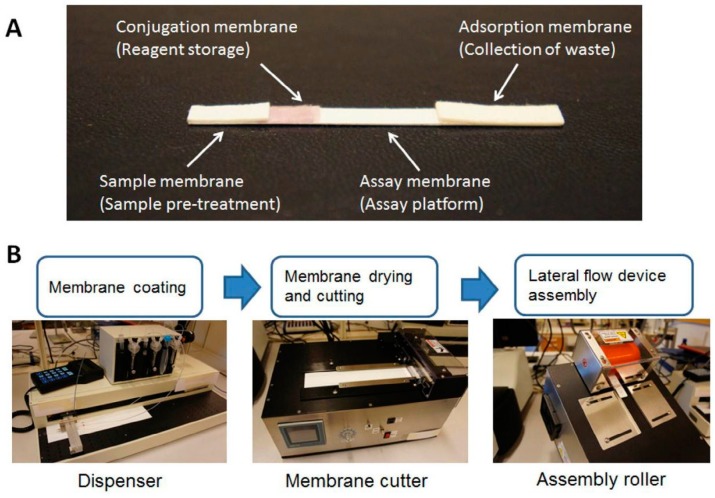
(**A**) Schematic diagram showing various membranes components and theirs functions in the design of the lateral-flow biosensing assay test. (**B**) Manufacturing processes for lateral flow tests includes: membrane coating, membrane drying and cutting, lamination and device assembly [38].

Based on the demands from POC, biosensing technologies have been advanced in the past few years. Among these newly developed technologies, visually-readable lateral flow strips are an inevitable evolution, and are considered to be the future of simple affinity assays [38]. Lateral flow assays are one of the most promising models of biosensors, with potential applications for the onsite detection of target molecules [39]. Lateral-flow strip includes several pads: a sample, a conjugation, a nitrocellulose membrane, and an adsorption pad. These pads enable the test to be completed through a sample preparation and collocation, bio-labeling, affinity assays, and signal generation and detection (Figure 6). Current trends in lateral-flow strip technology involve developing a portable and wearable device linked to the internet through smart-phone technology as an ideal POC approach. Current challenges in lateral-flow strip development are its relatively lower sensitivity, its degradation defects of electrochemical response, and its higher costs.

Visible colorimetric biosensing has a bright future in detecting nutrients, pathogens, and contaminants of foods because it is rapid, simple, highly selective, and sensitive [40]. The sensing technology is enabled through SPR, nanotechnology, thin film interference, and colorimetric arrays. Metal nanoparticles with their optical properties facilitate the visible colorimetric change leading to rapid detection when interacting with analytes, which avoid the needs of fluorescent and radiological labeling. Current challenges facing colorimetric biosensing are its label-free detection enabling, real-time analysis facilitation, single-step detection possibility, portability enabling, nanotechnology combination possibility, and higher costs.

Label-free, real-time, and paper-based affinity sensing based on SPR and quartz crystal microbalance (QCM) has been developed for food quality analysis [41]. This technology uses affinity receptors like nucleic acids, antibodies, and biomimetic receptors such as molecular imprinted polymers/aptamers (MIP). It can be applied to detect pathogens, endotoxins, pesticides, food origin, and genetically modified organisms. It can be used to further develop flexible POC devices by selecting various receptors targeting food issues to achieve the required selectivity and sensitivity; choosing suitable matrices as either onsite measurements or online monitoring, and designing as either a miniaturized or portable format.

Online monitoring techniques have been developed for control, surveillance, and optimization in food production, e.g., in downstream food bioprocessing [42]. In food bioproduction, the upstream processing measures for pressure, temperature, pH, and dissolved oxygen; on the other hand, the downstream processing focuses on removing adverse products and other impurities like host proteins and DNA. Online monitoring provides critical operation in ensuring product qualities, efficient production processes, and optimized economic effects. Soft biosensors are important in online monitoring techniques because they transfer signals from the online monitoring devices to mathematical models to enable information analysis. Current challenges in online monitoring are choosing suitable tools to identify quality attributes and process parameters and to meet regulatory demands, and higher initial investment costs.

Radio frequency identification (RFID) is a newly emerging technology in the agri-food industry [43]. RFID can be combined with biosensing, online monitoring, and other intelligent systems to increase information accuracy, to improve operation speed and efficiency, to minimize costs in labor and operation, and to reduce losses in inventory. It has been applied to food library management, theft prevention, food quality monitoring and control, animal tracing and control, and intelligent transportation. Current challenges in RFID are low awareness of benefits, various international standards, implementation issues, and high development costs. Efforts made and continuous requirements include the development of longer lifespan and lower cost RFID, combining it with artificial intelligence, and including biosensors with various functions.

Smartphone technology has been deeply integrated into biosensing technology; and is often employed as a displayer, analyzer, and controller for rapid and real-time POC monitoring [44]. Its application to biosensing particularly focuses on SPR, electrochemistry, optics, and near-field communication to develop light-weight, compact, and low-cost sensing devices. Its integration in sensing chips, test strips, and hand-held detectors reduces costs and simplifies the design in the biosensing systems. Current challenges in smartphone technology are minimizing optical components, advancing computational capacities to improve image qualities, developing microfabricated paper sensors with colorimetry under ambient illumination, and improving its cost effectiveness and portability. The future commercialization of smartphone-based biosensing devices is built on its real-time self-measurement of targeting analytes to fully explore this technique [45]. Researchers need to be motivated with a vision of smartphone biosensing of “one in every home” in the future.

The above contents, thus far, describe up-to-date concepts in POC technologies; multiple POC device candidates have been developed to meet these conceptual technologies. For example, a wearable biosensor in the form of a mouth-guard has been developed based on screen-printed electrode, Bluetooth low energy transceiver, and miniaturized instrumentation [46]. It is designed to monitor salivary uric acid levels non-invasively, a biomarker indicating multiple diseases such as diabetes, gout, and renal syndrome. It relies on real-time wireless information sensing and transfer to consumer electronics such as cellphones and laptops for diagnosis, processing, and storage. It is highly stable, selective, and sensitive in salivary uric acid detection with a detection limit of 2.45 mA/mM. This mount-guard biosensing platform can be further expanded to a sensor array for multiple salivary analyte detections with its circuit board miniaturization.

## 4. Biosensing in Food Safety and Security

The global population is expected to reach 9 billion by 2050, and will require 70% more foods, which poises an urgent need to food safety and security, and to the improvement of effectiveness and efficiency of food chain [47]. Food safety and security are related to all steps in the food chains from food farming, production, process, packaging, transportation, and all the way to consuming [48]. Hence, high-end food risk assessment and safety analysis with state-of-the-art technologies are of utmost significant. Biosensors represents a cutting-edge frontier in food quality and safety management at the forefront of the agri-food sector [49]. These technologies include aptasensors, microfluidics, and other biosensing technologies that can detect and manage food nutrients, pathogens, and toxins [50].

One of the advanced biosensing technologies that can detect both pathogens and toxins effectively are mammalian cell-based biosensors, which meet the current requirements in intensive and high-end food safety measures [51]. Another advanced tool is information technology providing mess data storage, process, and communication between food industries, retailers, stakeholders, and consumers, representing a more efficient food safety management system [10,52,53]. Biosensing technologies applied to the field of food safety and security have been developed for nutrients and qualities detection, pathogens detection, and toxin detection as described next.

### 4.1. Nutrients and Qualities Detection

The measures in food security can be divided into two categories: postharvest loss and food biosecurity [54]. Food biosecurity indicates food contamination and destruction by natural, political, unfair economic gain, warfare, or exacting revenge, which is covered in the later sections. On the other hand, postharvest loss indicates the nutrients and edible conditions in food which need to be preserved by technologies between the harvest time and the consumption moment. Since the time varies from minutes to years, the technologies focusing on postharvest loss reduction are significant in preserving and reducing the loss. Postharvest losses of food from different regions of the world are summarized by the Food and Agriculture Organization (FAO) of the United Nations as shown in Table 1.

Multiple causes are responsible for postharvest loss. For example, biological and microbiological factors may consume or damage food qualities; and these factors are bacteria, fungi, mites, insects, birds, and animals. Chemical and biochemical reactions may destroy or contaminate foods causing losses, which can include fat oxidation, the Maillard reaction, enzyme reactions, and lubricating oil or pesticide contamination. Moreover, mechanical causes like abrasion, spillage, and bruising; and physiological causes like senescence, sprouting, and transpiration or respiration changes in fruits and vegetables, may all damage and destroy foods or food components.

Modern technologies such as biosensing can be used to preserve food qualities and prevent postharvest loss. For example, biosensors have been developed to detect and analyze the amounts of sweeteners in foods, which can be used to detect both natural and artificial made sweeteners [55]. Sweeteners are used commonly in food processing and production; however, they have recently been identified as cause health issues in humans. A multi-channel biosensor has been designed to detect and analyze both natural and artificial sweeteners using electro-physiological sensing from taste epithelia. The signals are analyzed by spatiotemporal techniques to detect long-term signals from sucrose, glucose, cyclamate, and saccharin, respectively. The biosensor can differentiate between different concentrations from various sweeteners with dose-dependent increase responses by the taste epithelium. It can also differentiate between two natural sweeteners: sucrose and glucose, with two patterns of signals. The detection range for glucose is 50–150 mM, and for saccharin is 5–15 mM.

Multiple biosensors have been developed to measure glutamate levels in foods. Glutamate is a popular food additive in the form of monosodium glutamate to enhance umami flavor, and reduce salt intake [56]. However, it has been found to be harmful to human bodies and agricultural products. Even though glutamate is a natural neurotransmitter in our brain, at high concentrations it may induce neurotoxicity, causing damage to the muscles, kidneys, liver, and brain. Its release from human body can contaminate water sources to inhibit root elongation and seed germination in farming products. Since its concentrations vary in different food products, detection of glutamate is challenging. Current glutamate detecting biosensors incorporate either glutamate dehydrogenase (GLDH) or glutamate oxidase (GluOx) as bio-recognition elements. Biosensors comprising GluOx are simple compared to those involving GLDH because, the later involve NAD^+^ as a cofactor in the reaction. Current challenges in the biosensor development are that it requires higher operational potentials to oxidize NADH on electrode, high cost of enzymes and their low stability.

Another set of biosensor development in food safety focuses on detecting genetically modified organisms (GMOs) in food products [57]. GMOs had been considered a biotechnology revolution in all terrains of agricultural products since the 1990s. Thus far, more than 45% of soybean, 40% of corn, and 50% of cotton in the world are GM products; additionally, GM is also seen in livestock. However, recent studies indicate that GMO products may harm human and animal bodies through gastrointestinal issues, resistance to antibiotics, allergenicity, destruction of farm product diversity, and undesired gene flow to other organisms. Biosensors are developed to measure GMOs in foods and feeds through detecting GM genes using isothermal DNA amplification and rapid detection signal detection [57,58]. The major challenges in GMO detection is detecting unknown DNA genes which may be resolved by high-throughput technologies such as the combination of biosensing and arrays, and the establishment of GMO gene databases.

### 4.2. Pathogens Detection

Biosensors targeting pathogen detection such as bacteria (Table 2) and fungi (Table 3) began more than two decades ago because of their reduced format; one device to address several issues, and a multi-panel of signal detection [59]. In biosensor design for pathogen detection, ligand motif represents a critical element because it determines the sensitivity and efficiency of the device. The goal is to design a rapid, specific, and sensitive platform to detect the presence or absence of pathogens in food samples. It has been found that perfect ligand does not exist and various ligands have different advantages. Current challenges in pathogen biosensor detection are present in combining bioreceptors to detect a wide range of microbes in various samples; new synthetic ligand designs such as aptamers, small molecules, and peptides; and integrating of various ligands in a portable device to reach rapid, reliable, and low-cost detection.

In bacterial pathogens, *Salmonella* sp. is one of the most important foodborne pathogens because of their higher rates of food contamination and outbreaks in the world. Biosensors detecting *Salmonella* sp. have been focusing on electrochemical measures with simple and rapid features [60]. Great progresses have been made in phage-based separation and immuno-magnetic detection using either antibodies or DNA based nanotechnologies to facilitate simple and lower-cost detection without losing accuracy and sensitivity. However, in both academic and commercial development, none of them reach the stage of meeting all requirements in complex food sample measurement and the regulation limit. The future goals involve shortening the test time, identifying validation parameters, and enhancing autonomic and portable properties in *Salmonella* sp. detecting biosensors.

Bacteriophage-based biosensing is developed for detection of foodborne bacterial pathogens based on viruses infecting bacterial cells [61]. Bacteriophage-based techniques can be found in two types: unmodified lysing phages and modified phages. Unmodified lysing phages can lyse bacteria specifically and release specific components to facilitate detection, in which the modified phages carry reporter genes to facilitate the detection of their growth/amplification inside bacterial cells. Modified phage amplification tests have their advantages in detecting multiple strains of bacteria; and their critical element is the propagating strain as sensor cells, which are fast-growing and easy to measure. Currently almost no commercially available products exist for bacteriophage detection except one product from the U.S. market. A commercial phage detection product is provided by a U.S. business combining lateral-flow and bacteriophage to detect *Staphylococcus aureus*, and it has been approved by the U.S. Food and Drug Administration (FDA). The product is applicable to both clinical and food samples, and has been developed in a biosensing format to enhance its application and to reduce its costs.

Microbial fuel cell sensors can be developed as an early warning system in food production because it carries a self-sustainable power source without the needs for additional sources in long-term monitoring [62]. It avoids multiple bottlenecks and limits in pathogen detection in food products. One of its significant applications is to monitor both toxicity and biochemical oxygen demand in water quality because its self-powered sensing allows for onsite and online monitoring. Future research should focus on improving its sensitivity in detection range and limit, decreasing its recovery time post-biofilming, and combining detection algorithm, kinetic, and empirical models to differentiate between toxicity and biochemical oxygen demands when they occur simultaneously.

Besides bacteria, fungi infection and contamination in the food chain are considered significant, particularly in plant food production, where it may lead to toxic metabolites and mycotoxins appearing in foods, affecting human health, food security, and food export markets [63]. To effectively monitor and control bacteria and fungi in food chain without mitigating the environment, a concept of sustainable intensification (SI) system is recommended in agri-food [64]. The SI concept is to produce more from the agri-food system, with fewer inputs and without negative impacts on the environment and resources. The key elements in an SI system are in reducing reliance on nutrients and agrochemical inputs, enhancing biodiversity, increasing resilience to biotic and abiotic stress, and maintaining the ecosystem and environment.

### 4.3. Toxin Detection

**Figure 7:**
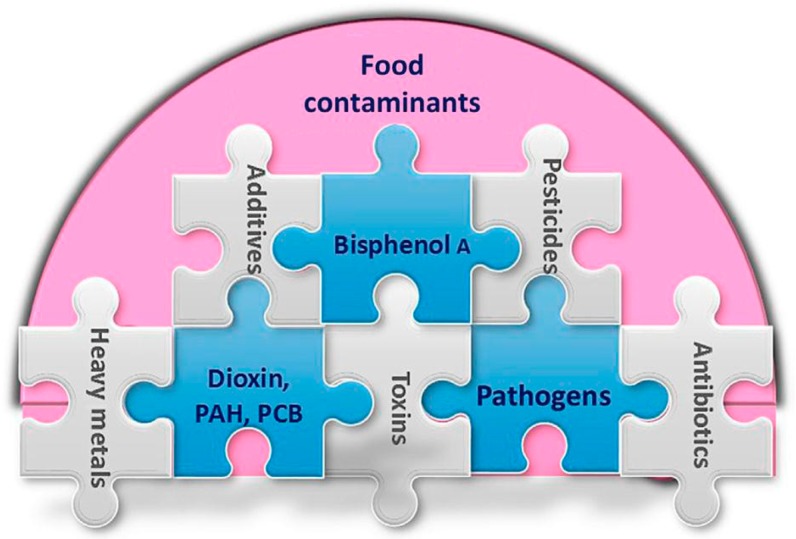
Predominant food contaminants and the target analytes in the food manufacturing industries [67].

Electrochemical biosensors for fast detection and assessment of food toxins belong to the main stream of development in food safety [65]. Various platforms have been designed to allow customized and individualized devices to meet the specific requirement of situations and environment [66], and to reach a detection limit of nM to fM levels [35]. For example, bioreceptor arrays address individual electrodes functionalized with various bioreceptors with binding targets to facilitate unique binding profiles. Besides electrochemical biosensing, other biosensors like optic and piezoelectric sensing have been applied to toxin and chemical detection in food production (Figure 7) [67]. Fluorescent nanoparticles have been developed to sense toxins in foods and corps including on-surface, and inter- and intra-cellular of foods [68].

One of the key challenges in developing a fully automatic toxin detector is toxin extraction from complex food samples [69]. Future systems are expected to extract, process, and measure toxins to determine their harmful levels from food and water samples automatically [69,70]. Advanced separation techniques have been coupled with SERS in identifying, discriminating, and quantifying chemical toxins in food matrices [71]. Furthermore, chemical contaminants from food processing can be a challenge even though they are usually in lower amount [72]. Another challenge in food toxin detection is its lower stability, selectivity, and sensitivity in which MIPs can be a solution to offer stable and low-cost alternatives [35].

Heavy metals, such as Ag^+^, As^3+^, Cd^2+^, Hg^2+^, Pb^2+^, and Zn^2+^, are classified as chemical contaminants form stable oxidation states and interfere with the metabolic pathways, leading to health problems [73]. Biosensors with aptamer and DNA-based properties can detect heavy metals at nanoscale levels, as well as on a very large scale, which are suitable for food safety screening and monitoring. A heavy metal detecting biosensor is based on genetically modified bacterial cells and a green fluorescent signal amplifier to detect arsenite in foods [74]. Its arsenic detection lasts only one hour with a detection range of 5–140 µg/L of arsenic and can be integrated with optical power output for its optical fiber biosensing future. Other biosensing technologies such as aptamers, nanoparticles, and graphene electrodes have been applied to arsenic detection and evaluation successfully with possibilities to be developed as rapid, simple, easy-to-use, and low-cost devices [75].

Nanotechnology has been applied to two different fields of pesticides in agri-food: as a pesticide delivery vector to achieve pesticide management and as a pesticide trace-amount detector. In the first field, nanoparticles can modify pesticides to target insect pest slowly, which helps to prevent pollution of both ground water and top soil, reduce pesticide amounts, and increase efficiency [76]. In the second field, nanotechnology based on bio- or biomimetic molecules such as antibodies, enzymes, aptamers, and MIP-like macromolecules to increase stability, selectivity, sensitivity, and detection speed [77]. In addition, whole cell-based biosensors applied in pesticide and herbicide detection include bacterial, fungal, algal, and mammalian cells; and they assist in the development of rapid, accurate, real-time, and cost-effective tools in decontamination procedures and damage preventive casualties [78,79].

Other toxins significant in food production include carcinogens, odorants, and marine contaminants. Carcinogens represent a complex group of trace-amount toxins such as pesticides, heavy metals, mycotoxins, and acrylamide, in which the challenge is the difficulty in detecting trace- amount toxins; and imprinted aptamers, nanotechnology, and biosensing are hopeful for future promising application [80]. Odorant binding proteins are sensitive and soluble molecules useful in odor detection for olfactory animal systems. A nanosensor has been developed, combining localized SPR and small odorant binding proteins from honeybees, in which the detection range is 10 nM–1 mM using a quantitative nanocup array [81]. Marine contaminant detection is used to monitor and maintain a healthy environment for marine food systems. Lastly, biosensors can be applied to marine food safety through their features of sensitive detection, miniaturized devices, wireless communication, and small-scale network to be developed as advanced analytical and monitoring tools [82].

### 4.4. Miscellaneous Compounds Detection

There are two major routes through which miscellaneous compounds enter into food products. Among them, the compounds used during the farming and agricultural activities are alarming and require major attention. Some of the most notable antibiotic residues in food products are chloramphenicol, tetracycline, erythromycin, tetracycline, penicillins, ampicillin, quinolone, and cephalosporins. These antibiotic compounds are used beyond permissible levels as a growth promoter and to increase animal muscle mass. They cause rare and serious health problems such as antibiotic resistance in humans and antibiotic resistant pathogens. Much effort has been made in recent years for the detection of antibiotic residues in food products through biosensors [83,84].

Another important aspect is the steps involved in food processing itself. Most of us think that food processing steps are essentially safe, but in certain instances this is not the case. With the discovery of acrylamide in processed foods, the issue is seriously being analyzed by various health agencies and researchers [85]. Some of the processing contaminants are acrolein, acrylamide, advanced glycation end products, chloropropanols, glycidols, furan, heterocyclic aromatic amines, hydroxyl methyl furfuryl, and polycyclic aromatic amines from thermal processing. During the fermentation process, contaminants such as acetaldehyde, biogenic amines and ethyl carbamate are produced. Preservatives and additives which are added to food products to improve their sensory properties and shelf life, transform into contaminants. Among them, benzene, methylimidazoles, nitrosamines and semicarbazide are notable ones. Novel processing techniques developed as an alternative to thermal processing were also found to produce certain unnecessary compounds such as alkylcyclobitanones, free radicals and furan. The list is too exhaustive to be discussed in this section, with lot of new compounds being added frequently [9,86]. Biosensors for most of the compounds have not been reported to date; however, some of the biosensors for food processing contaminants are discussed in the following references [9,23,87].

Adulteration is a deliberate attempt by the food producers and processors to add components of low cost to mimic the food through their sensory and physicochemical attributes. The definition for food adulteration varies geographically and there are strict regulations in place to prevent food adulteration. However, with motivations by monetary benefits, it is a serious issue for basic food products in underdeveloped and developing countries. It is also a serious issue in gourmet food products in developed countries. In recent times, advanced biosensor and molecular techniques have been developed for the detection of food adulteration [88,89,90].

## 5. Biosensing in Food Packaging and Supply Chain

In recent years, active and intelligent food packaging technologies offer sustainable safety and quality of foods, real-time monitoring packaging process, and improved shelf-life to meet increasing demands from manufactures and consumers [91]. In food packaging, nanomaterials are incorporated for biosensing, increase shelf life (antimicrobial properties), and intelligent and robotic technologies to educate and alert the consumer for food safety and quality. This section reviews biosensing technologies in food packaging and the supply chain separately.

### 5.1. Food Packaging

**Figure 8:**
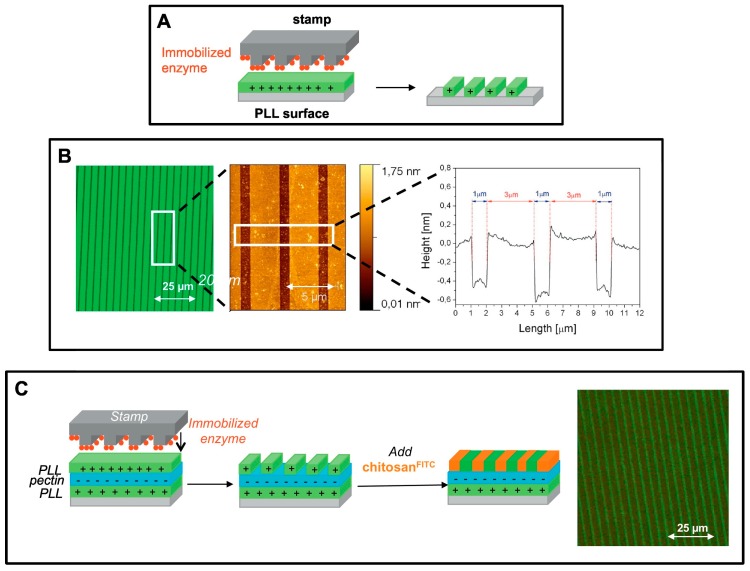
Nanosensing application in food packaging. Schematic representation of the enzymatic microlithography process with (**B**) the resulting Atomic Force Microscopy (AFM) topographic image of the patterned Poly-L-Lysine (PLL) surface (scale: 5 μm) and line profile of the corresponding AFM image. (**C**) Patterning process of multilayered thin films composed of pectin and PLL. When the stamp is applied on the film, only the top layer is patterned, which allows the formation of alternate positive and negative lines highlighted by further site-selective adsorption of cationic polymer [93].

Nanotechnology demonstrates its potential in food packaging and quality assurance to reduce the ecological footprint to the environment, and to provide healthy foods to consumers [92]. Nanotechnology can be applied to the combination of antimicrobial and antifouling, food protection from moisture and oxygen, detection spoilage, and monitoring storage conditions. Titanium dioxide, confirmed to be non-toxic to humans, can be applied to nanotechnology food packaging because it is a food additive with a role in food perception (Figure 8) [93]. However, its effects post digestion and adsorption into the body require further studies. Cellulose nanofibers, as a biodegradable and bio- renewable nanomaterial, belong to a hopeful candidate worthy of further exploration in food packaging to decrease costs and reduce environmental impact [94]. The main concerns in cellulose nanofibers are their suitability, sustainability, and limitations in food packaging [95].

Nanodiamonds have been found with antibacterial and anti-inflammatory properties and a future possibility of application in food packaging [96]. Nanodiamonds may be used as biosensors and food additives in packaging to protect foods from being spoiled by microbial and toxins. Nanodiamond particles in food packaging have been shown to improve flexibility, durability, and humidity and temperature resistance; and maybe also improve antimicrobial and anaerobic conditions. The overall challenges in nanotechnology and food packaging are its possible adverse effects on human health, its short and long-term effect on the environment, and nanomaterial specific laws and regulations.

The next-generation of food packaging relies on intelligent and robotic technologies. Intelligent food packaging systems may detect, sense, and record changes in food products, their package, and their environment to ensure food qualities [97]. The systems meet the requirements from the conventional food packaging, and can turn all of them into the next generation of advance; and these requirements are food protection, package communication, convenience for food consumers, and food containment. Currently available intelligent and robotic technologies in food packaging are relatively early and immature, such as the cradle-to-cradle (C2C) and cradle-to-grave (C2G) sustainable intelligent food packaging systems. These novel technologies are need to be examined for their properties, hurdles, benefits, and adverse effects on food qualities. Undoubtedly, intelligent and robotic technologies and their application to food packaging will shed lights on better food quality control, better food monitoring, and safer and higher food qualities.

One of the significant application of intelligent and robotic technologies in food packaging is as anti-counterfeiting devices and anti-contaminant sensors [98]. In this sense, nanotechnology can be combined with intelligent and robotic technologies in food packaging to achieve the goal of meeting consumers’ demands for healthier and safer food products. The integration of nanodevices and nanosensors into food and beverage products provides anti-counterfeiting and securer properties in consumer warning and reminding applications. As such, the application of these technologies in food packaging may enhance the reliability of food products and increase consumer confidence of food safety in the near future.

Current developed intelligent devices in food packaging are present in the forms of labels, dots, tags, and inks, which bear different functions in ensuring food qualities and safety [99]. Food quality sensors, indicators, and non-sensor components can be combined with and integrated into packaging to monitor food condition and warn consumers about food freshness and spoilage [100]. The application of nanotechnology in food monitoring reduces foodborne diseases, decreases food waste, and reduces food product deterioration and spoilage.

Challenges in the application of intelligent and robotic technologies in food packaging are awaiting resolution [93]. For example, the costs of the novel technologies are estimated to be 50–100% of the whole final package. Since the commonly acceptable food packaging fee should not exceed 10% of the total good cost, intelligent and robotic technologies may make the foods less affordable and result in a negative cost-benefit ratio. Another challenge is the complexity of intelligent and robotic systems which may be resolved by combination of various components in food monitoring and control to simplify the material and devices used. An additional challenge is developing reversible, reusable, and long-lasting systems to replace current irreversible, disposable, and single-use devices.

### 5.2. Supply Chain

Theoretically, the food supply chain should be able to provide adequate information to consumers and other concerned bodies about food attributes, animal welfare, GM issues, and country of origin. In this regard, food traceability system is ideal in fulfilling the requirements because issues currently exist in food traceability [101]. Food traceability system is considered to be a part of food logistics management; its full chain development being complex in nature; and deep understanding being required in technological, social, legal, and economic issues. Future development should focus on its technology advances, its linkage to food production, its integration with food logistics, its standardization of information exchange and data capturing, and its effective communication with stakeholders and consumers.

A conceptual framework of food supply chain assessment and logistics of food products targets the following approaches: food quality, food safety, food sustainability, and logistic efficiency of food process and its products, which covers the whole food supply chain from the “farm to fork” [102]. Issues in this field are that quality status of food products often stops at the producers without tracing strategies until they reach consumers; this needs to be changed because food status changes according to its exposure to heat, lights, pressure, mechanical shock, and vibrations. In this case, counterfeit integrated circuits technology is applicable and effective; however, expensive RFID-based solutions in supply chain systems control and product traceability monitoring. Even though most current food enterprises are interested in such technologies, none of them can afford such an expensive food supply chain traceable system. Effective and inexpensive systems with flexibility, closed-loop, and remote-control features are highly expected in this market.

Two technologies are current predominant in food supply chain traceability field: RFID technology and information and communication technology (ICT). RFID technology is promising in tracking, monitoring, tracing, and improving food qualities in its supply chains [103]. Multiple international food enterprises are applying RFID technology to enhance food quality visibility from farm to fork with amazing consequences, and is expected to continue growing in the direction of improving performance and reducing costs. On the other hand, ICT-based technology is widely applied to supply chain solutions, such as supporting food product traceability, maintaining electronic records, capturing related data information, and sharing traceability attributes [104]. ICT solutions are useful in information communicating about food inputs, food processing, GM background, food related diseases, pest tracking, and environmental measures.

Pervious research into food supply chain traceable systems has mostly focused on traceability at the retail point, ignoring points beyond the retailing and consumer portion. Even though it comes with a cost, traceability to reach the consumer segment is particularly important because the costs can be significantly higher with unsafe foods at the retailer, consumer, and governmental levels [105]. It is time for all the parties to promote a novel reality of food supply chain traceability systems. Current challenges in food supply chain assessment and logistics are correlation measurement between logistic regulation and decision, food status monitoring and evaluation, and safety measures at food consumption points. Future research should target the integration of food logistic information such as packaging solutions, container loading, transportation modes, delivery planning, and storage conditions, which should be combined with food quality, perceptions of food safety, and food environmental effect [106].

## 6. Biosensing in Food Waste Processing and Environment

### 6.1. Food Waste Processing

**Figure 9:**
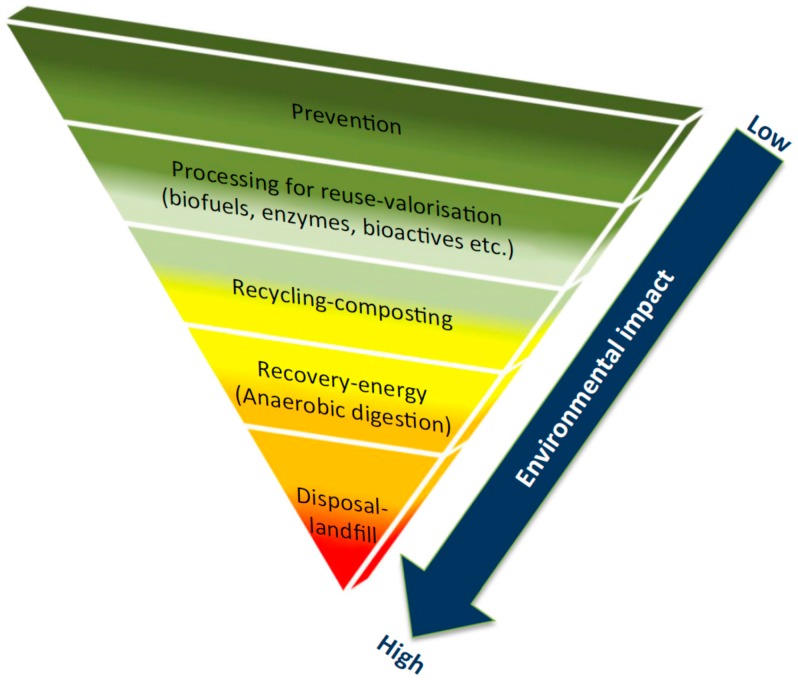
Hierarchy for handling food waste processing: Effective strategies and technologies in food waste management [107].

The generation of food waste is on its rise due to a growing global population leading to increased food production and consumption, which has been intensified because of ineffective and slow processing of food waste [107]. Development of effective strategies and technologies in food waste management, treatment, and disposal are urgently needed. Food waste is a complex reservoir ranging from raw materials to important commercial metabolites, such as proteins, carbohydrates, nutraceuticals, and lipids. Among the currently developed technologies, nanomaterials, bioactive compounds, biofuels, biodegradable plastics, and enzymes are the main focuses, in which enzyme-related studies have been most focused upon (Figure 9).

Food waste in water represents a unique field compared to solid waste; and biosensing, nanomaterials, and wireless communication have been introduced in this field to enhance the accuracy, power consumption efficiency, data storage capabilities, communication ability, remote sensing, and commercialization [108]. Biosensing technologies can be applied to test-treated waste water released from local waste water treatment plant, because it is important to monitor the treated waste water to prevent harmful effects and reduce adverse influences on the local environment and organisms living in water, such as fish and other species [109]. Future challenges in water waste biosensing processes exist in establishing multitude of sensing arrays with multi-location deployable, unique sensing properties, and better sensing accuracy; developing wireless networks to improve remote sensing qualities, and improving cost-benefit ratios in emerging waste processing sectors.

Enzymes from microbes including bacteria, fungi, yeast, and actinomycetes, intracellularly or extracellularly, have a wide range of commercial applications in food industry. The industrial enzyme market globally totaled $3.3 billion in 2010 and is expected to rise to $5 billion by 2010 [110]. The unique properties of enzymes, such as fast action, high specificity, and biodegradability, make them ideal in industrial processing, with mild reactions and lower waste generation. Enzymes can be used in their natural format, but they are often modified to enhance their properties. For example, they can be modified to be immobilized on clay minerals to bear potentials in biosensing and biocatalysis of food waste [111]. The combination of non-covalent enzymes, clay minerals, biosensing electrodes, and nanomaterials has been proven to be effective in processing solid and water waste in food production.

One of the hopeful future enzyme strategies is biocatalysts derived from seafoods such as chymotrypsin, fish trypsin, and cold-active lysozyme/chlamysin, and they among others are already commercially available [112]. These seafood derived enzymes have the potential to be genetically modified to improve the limitations in their industrial application, because most of these biocatalysts are limited by their natural catalytic properties and source quantities, despite the fact that they are available in huge quantities as discards and wastes. Future research should target the combination of proteomics, genomics, and metabolomics to improve seafood enzyme production quantities, enhance catalytic functions, and increase enzyme purity to simplify downstream processes [113]. New opportunities are open for the aggressive development of seafood biocatalysts in food waste industry; yet, there is a long way to go before the realization of their practical applications occurs.

### 6.2. Environment Protection

In current food production, contamination sources exist in agriculture chemical use and other underestimate origins, which call for urgent development of biosensing instrument to assess and control food qualities and to reduce potential hazards [36]. The current situation is that none of the single functioning biosensors can take over the complex tasks in food contamination measure, but a combination of various technologies is required, such as the combination microbial biosensors, DNA biosensors, biochemical DNA repair, and metabolic activation of toxins like carcinogens. This multisensing approach integrating a couple of biosensors on one single platform with similar recognition targets is the most useful and hopeful trend in identifying chemical pollutants and improving detection signals.

Nanomaterial such as gold nanoparticles, quantum dots, carbon nanotubes, and nanowires have been applied to food contamination field to assess, monitor, and control food environments [114]. The technology provides tools in food security to identify and prioritize environmental risks and reduce food contaminants while balancing the ecosystem. For example, nanotechnology has been combined with liquid photo-purification techniques in assessing, managing, and controlling high-quality milk products, which replaces conventional milk pasteurization with eco-compatible green solutions. In addition, the application of nanoencapsulation technology in food waste processing occurs as a collaboration between scientists and industrial groups, which offers optimized extraction and healthy formulation of certified food products.

Another issue facing food production environment is its impact on climate change, and agriculture activities are responsible for releasing at least 17,000 megaton of carbon dioxide to the atmosphere annually [115]. The topic of how to reduce negative impact and increase positive effect from agriculture on the environment is popular nowadays. One of these efforts is biofuel production from microbes as the future of the third generation of fuels overcoming most disadvantages and enhancing and creating novel advantages. In this sense, biofuel generation from microalgae-bacterial interaction is confirmed to be one of the promising approaches due to their advantages, such as smaller sizes, higher oil content, better growth, and lower costs [115]. The microbial fuel-generating cells can produce electricity through oxidation of inorganic or organic waste, such as brewery wastewater and orange peel waste. With the assistance of genetic modification, proteomics, transcriptomic, omics technology, and metabolomics, biofuel production from microbes, plants, and algae represents an emerging future with greener potential and ecosystem-friendly possibilities.

The above advanced technologies in waste and environmental management require higher agricultural plant production and this increases loading on already strained plant needs. Effective strategies are urgently required to maximize plant community performance through preventing and inhibiting intruders such as invasive plants, herbivores, pathogens, and mutualists [116]. Related research should focus on several key aspects in enhancing plant performance and production. First, multiple resources should be considered instead of single resources such as simple and identical plant vegetation, because introducing multiple traits can enhance plant production and competition for water, light, and CO_2_. Second, crop specific growth model should be developed based on specific sites and specific species, because it assists crops growth under suitable conditions with elevated CO_2_ effects on crop functioning and structure. Third, phenotypic plasticity should be included to treat environmental reactions but not trait values; this allows plant-plant interaction and their dynamic architectural train interplay. Fourth, selective effects from agricultural practices should be analyzed to assess their impact on crop functioning to reflect changes from climate, production volatiles, parasitic weed composition, and the scale and density of plants.

To ensure plant growth and production, soil management has never been so significant. Current soil biodiversity is under threat from the use of chemicals and fertilizers; unfortunately, soil biodiversity determines plant biodiversity [117]. Soil biota govern soil organic matter formation, turnover, and nutrient storage and cycling; and higher soil diversity is significant for soil functioning. It can be used as indicators in sensitive measures of adverse effects derived from artificial components and adverse agricultural activities, as well as shifts in nutrients and carbon cycling. For example, based on research conducted on soil diversity, it is recommended that less intensive agriculture like organic farming should be performed, because this reinforces soil diversity and its self-regulating status. Nevertheless, soil diversity research is in its infancy stage and challenges need to be overcome in soil biota presence and status, longer term soil biodiversity studies, and biogeographical distribution of the diversity. This research hopes to reveal options in agricultural sustainability, plant production maintenance, and restoration of soil qualities.

## 7. Biosensing in Food Quality Assurance

The key value in food industry, as a complex and global enterprise, is in ensuring high food quality because it is the heart and soul of foods consumed by its customers. However, this industry needs to adopt novel technologies besides automobiles and electronics, to meet requirements in ensuring food quality, in which biosensors based on nanotechnology and other novel techniques are significantly critical [118,119]. One of the issues in biosensing technology of food quality is the potential toxicity of biosensing materials; and the challenge is in exploring organic and non-toxic biosensing materials to ensure that they are not harmful or less harmful to food products while food quality is assessed. To tackle this challenge, multiple studies have attempted to develop bio-organic sensors offering biocompatible and biofunctional materials in designing smart sensing devices. Thus far, these organic materials, such as protein, DNA, cellulose, silk, waxes, and biodegradable aptamers, are in their laboratory stages [120].

Graphene-based material and its derivatives like graphene oxide and its reduced formats have been introduced to the biosensing of food quality and its assessment [121]. They bear superior properties in optical, electrical, thermal, mechanical, and chemical absorption, which make them favorable for food industrial application. The generation of novel graphene materials from foodstuffs and their application in food quality analysis and detection of organic compounds, food composition, toxins, and contaminants ascertain high food quality. Graphene materials with anti-bacterial and antibody-functionalizations have a promising future in this category.

Electrochemical aptamer based biosensing offers unique properties like rapidness, specificity, sensitivity, portability, simplicity, and lower cost compared to conventional measures [122]. Its commercialization faces several challenges including a bottleneck in sample preparation, technical issues in introducing nanomaterials, qualified comparison to other technologies, and development of aptasensors detecting additional food quality-related issues. Magnetic nanomaterials and their integration into microfluidics pave ways to better sample pretreatment and rapid, simple, and easy-use formats in food quality analysis [123]. Major challenges in food quality biosensing technology include designing high complexity components to increase sensitivity, enhancing stability of immobilized receptors to facilitate commercialization, and fabricating multiplex portable biosensor with nanomaterials, automatic properties, and low-cost formats.

Electro-chemiluminescence biosensors enable possibilities for designing highly sensitive and complex devices without increasing costs [124]. It is a powerful analytical technology that is applicable to food quality assessment with promise in future commercialization because of its outstanding performance. The advantages of electro-chemiluminescence biosensing are that it can be easily integrated with other popular techniques like nanomaterials, aptamers, immunoassays, and microfluidics. It has been applied to multiple food quality measures such as pesticides, toxins, residual drugs, heavy metals, bacteria, and viruses. Its future target of development is in improving sensitivity in complex food matrixes to yield devices with miniaturization, rapidity, and affordable costs.

Microfluidics and their derived analytical devices are capable of detecting allergens, foodborne pathogens, residual pesticides, heavy metals, toxins, additives, and other contaminants in food quality management [23]. It bears the possibility to be integratable with other novel technologies to facilitate its development into a device with reduced sample amounts, increased portability, and enhanced miniaturization for field use in areas with limited resources. It can be designed as a platform integrating with aptamers, SERS, and computerization with a hopeful future of developing into devices with POC properties.

A novel application of nanomaterials in food quality assessment is nanoproteomics and its application in measuring qualities in foods and beverages [125]. Omics represents a high throughput approach that is useful in exploring suitable biomarkers for applications in foods and beverages to address safety, quality, authenticity, and technology issues. Nanoproteomics is an integration of proteomics and nanomaterials to facilitate acquiring vast protein information through proteomic profiling derived from various foods. Nanoproteomics can be further combined with SERS techniques and developed into a promising device with label-free, miniaturized, and high sensitivity properties. It can also be fabricated in a lab-on-a-chip format that is useful in diagnostic purposes, focusing on either increasing cell numbers or concentrating sample volumes while maintaining its overall sensitivity.

Multiple other biosensors have been developed in food quality assessment and management. For instance, a biosensor has been designed with pendant anthracene units and an on-off or off-on feature based on water soluble biocompatible oligoaziridine to detect toxic chemicals [126]. A sensitive biosensor has been fabricated using laser ranging and laser scanning with highly accurate state-of-the-art remote sensing in crop height and vitality management [127]. A soil nitrite biosensor has been designed using a nano-lipid platform with properties of optimizing physical and chemical variables from simulated soil and real samples, with a detection limit of 2.1 μg/L of soil extracts and 1 ppm of lowest field nitrite [128]. Biosensors can also be applied to measure live food sources such as live fish quality, freshness, and stress through image qualification and stress-related chemical (glucose) measures [129,130]. Furthermore, an optical capsaicin biosensor has been developed using immobilized 3-methyl-2-benzothiazolinone hydrazone hydrochloride hydrate (MBTH) on hybrid film and horse radish peroxidase (HRP) binding to chitosan film, to determine chili hotness with a detection range of 0.2–4.0 mM and a detection limit of 0.17 mM [131].

Robotic applications in agricultural food production are perhaps the most advanced technology thus far; and they combine biosensors with drones to monitor soil erosion, land change, air quality, illegal poaching, and wildlife in the field [132]. Future targets in robotic biosensing are the integration of artificial intelligence, monitoring of live food quality change, education and training of farming users and multi-stakeholders, and establishment of proper legal frameworks.

## 8. Biosensing in Food Engineering

Food engineering is a multi-disciplinary field combining physical sciences and product properties to generate processes and equipment, which converts raw agricultural materials and ingredients to convenient, safe, and nutritious food products [133]. Contemporary food engineering is facing challenges from modern knowledge and technologies in how to apply and integrate nanomaterials and computational material science to developing novel products and processes [134]. The ultimate goals are to generate food products with improved safety, security, and quality. Food engineering in process intensification focuses on improving flexibility, efficiency, and the sustainability of food production [135]. The values in process intensification are capital reduction to smaller and cheaper scales, reduced volume with higher safety, energy consumption reduction, and environmental protection. Process intensification attempts to improve the yields in raw agricultural materials, to enhance mass and heat transfer, and to accelerate component separation, mixture, and reaction. Contemporary food engineering influences food production critically as it faces social accountability, government oversight, and increasing global competition.

Food security and sustainability are facing challenges from politics, demographics, and economics. As defined by the United Nations, sustainability represents a better quality of life offered to people, and sustainability development is on a growth according to the World Commission on Environment and Development [136]. Food sustainability is dynamically linked to social, ecological, and economic issues aiming to meet the current and future requirements from the society, in which the demand is based more on socio-economic but not environmental changes. Accordingly, food policies from international agencies and governments are required to target not only secure and equal food access, but also a pacified and safe world [137]. This means that adequate food access is essential for a safe society, which includes making resources available to vulnerable and poor populations, and reducing gender and social discrimination.

Political activism never ends in policies related to food production. For example, in one of the most intensive food debates, GM products are the greatest concerns. While restaurant chains claim that they no longer carry GM products, implying that something is wrong in these products, pirate GM seeds are used in impoverished areas to feed families in need [138]. Similar political reactions and activities will never end, with emotion and impact on future food development, particularly with food engineering. The consequence is a higher and more intensive demand on the safety and security evaluation of engineered food products, with related studies being required to be conducted to safe-guard engineered foods and to reduce concerns.

One of the food production fields receiving little recognition in food engineering is soil organisms, which are ecosystem components with significant impact on food sustainability. Since soil diversity determines food diversity, soil ecological engineering is a novel concept in enhancing better soil diversity, serving the needs of increasing human populations, and reducing environmental impact through engineering soil organisms [139]. Away from the conventional trend solely focusing on increasing food production without considering sustainability, soil organism engineering focuses on the combination of soil biology and advanced technologies. It is based on state-of-the art knowledge on precision agriculture, and the concept that soil organisms are critically important in optimizing water and nutrient addition while reducing external use of resources. In this sense, soil ecological engineering results in increased soil diversity, effective land use, minimized yield gaps, and manipulated soil biota that use the limited soil source in a more efficient and sustainable way.

Bioengineered nanomaterials and their application in food production are in their infancy with pending issues because of their unknown properties on human health. As a new source of analytes, bioengineered nanomaterial technology integrates physical information such as shape, size and aggregation with chemical condition such as mass, concentration and composition [140]. Due to these complexity, the analysis and control of bioengineered nanomaterials are currently under development and facing challenges. Their analysis and control require a combined application of both sophisticated and novel technologies and instrumentation, including X-ray, optical microscopy, inductively coupled plasma mass spectrometry (ICP-MS), matrix assisted laser desorption ionization- time of flight mass spectrometry (MALDI-TOF-MS), and field-emission environmental scanning electron microscopy (FEG-ESEM) to provide specific and sensitive solutions. The complex processes of analysis and control in bioengineered nanomaterials also indicate an urgent requirement of standardizing nanomaterials and their use in food production industry.

Innovation in food sustainability is urgently required to ensure food production to overcome burdens from an increasing human population, nutrient use inefficiency, drought tolerance, resistant diseases, durable pests, and environmental issues [141]. As such, novel technologies like proteomics need to be extended to reach conventionally rare foods to enlarge food variety and availability. Those foods not considered in the conventional diet, such as cereals, potatoes, legumes, and lotus, are attracting researchers because they may survive unfavorable conditions from the environment. Furthermore, systems metabolic engineering sheds its light on increasing the development of microbial factories to produce engineered energy, chemicals, and other rare materials [142]. Future research should focus on precise genome-level metabolic engineering with signal network and integrated regulation, enzyme and pathway designs with better accuracy for non-natural chemical production, development of better prediction tools in protein structure, and fabrication of genome manipulation tools on a large scale, with convenient, efficient, robust, and multiplex properties.

For example, metabolic engineering can be applied to develop flavonoid detecting biosensors because flavonoids have been considered a significant nutrient with antibacterial, antiviral, antioxidant, immunosuppressive, and anticancer properties [143]. The engineered biosensors are designed based on transcriptional activator FdeR, auto-fluorescent labels, and *Bacillus subtilis*-derived Quantum dot regulator (QdoR). They show a 7-fold increase in fluorescent signal, resulting in linear correlation from various flavonoid concentrations. The detection range is 0.005–0.1 mM of naringenin, a key flavonoid pathway intermediate. This flavonoid bioengineering detection technique is also promising for development into flavonoid producing bacterial strains in the near future.

Bioengineering has been also applied to paper-based biosensors and chemosensors to enhance their simplicity, performance, and affordability due to the fact that these sensors are mostly cheaper, useful, and rapid with a POC future [144]. Paper-based sensing has been widely integrated into other novel technologies including genotyping, microfluidics, and nanotechnology; and it challenges other material based sensors like glass, silicon, and polymers. It is promising that paper-based sensing together with the benefits from bioengineering will go beyond its previous limits as a screening tool to become a critical alternative in affordable field test for environmental control, self-monitoring in food safety, and rapid onsite diagnosis.

Opportunities and challenges currently co-exist in food engineering as it sheds light on 21st century expectations and requirement [145,146]. Major challenges in food engineering have been identified as open innovation, virtualization, modeling, and social responsibility. Its open innovation indicates that the roles from academic research need to be revised in its creativity, employability, and intellectual property models to act practically as university start-ups. Its virtualization involves the application of virtual technologies, such as dedicated software, big data, cloud computing, and other breakthroughs to generate realistic benefits. Its modeling suggests a shift from empirical to physical based food production models, with applications from biosensing, nanotechnology, and other novel techniques. Lastly, its social responsibility requires that all food engineering processes should consider their impact on health and wellness, food complexity and uniqueness, consumer expectation and needs, and sustainability.

## 9. Current Progress, Solutions, and Future Challenges

Major challenges from food sustainability, based on current overviews from the literature, focus on three fields: nanomaterials and their application in sustainable agriculture challenges, energy sustainability challenges, and commercialization of sustainable technology challenges. These three challenges are revisited as follows.

Nanotechnology, as a leading application in agricultural monitoring and control, has multiple beneficial properties and applicability. It can enhance food safety and quality, enrich nutrient absorption from soil, reduce agriculture inputs, and increase the possibilities in the miniaturized device dimension [147]. It has been applied successfully to precise farming technology, intelligent feed, food waste reuse, agro-chemicals like nanoherbicides, nanopesticides, and nanofertilizers, and labeling and packaging, as well as multiple other agricultural fields.

The application of nanotechnology on food sustainability is expected to yield several consequences in the near future: combined nanomaterial and activated charcoal to enhance antimicrobial properties, food grade nanoemulsion used in fruit juice, integrated nano-microbials as water purifiers, efficient nutraceutical nano-delivery, and enhanced plant extracts conjugated with nano-packaging [148]. A predominant focus of nanotechnology is its application in precision agriculture, in which plant extracts from flowers, leaves, roots, and stems from diverse species have been integrated into nanomaterials successfully [149]. Nanomaterials enhance green synthesis in a single step with ion and metal reduction consequences, which is promising for room temperature usage, easy-use, modifiable and scale-up-able, and eco-system friendly. In green synthesis, co-enzymes and soluble metabolites such as phenolic compounds, alkaloids, and terpenoids are all reduced to nanoparticles. As such, nanoparticles are called “magic bullets” resulting in enhanced plant growth, site specific delivery of nutrients, and increased plant disease resistance.

The most significant challenges in nanotechnology are in the establishment of reliable rick-benefit assessments with standardized evaluation and processes; establishment of reliable and standardized methods in nanomaterial quantification, characterization, and evaluation of their impact on human and the environment; and engagement of all related stakeholders including farms, food industrial agents, non-governmental organizations, and consumers in a dialogue of public support and consumer acceptance [150].

Sustainable energy challenges can be addressed effectively by applied biological solutions. Several major applications have been explored in applied biological energy generation [151]. First, biofuels can be generated, stored, and renewed as bio-electricity to reduce the cost of solar electricity significantly, which can be achieved through leveraging by H_2_ or electron uptake driving carbon fixing metabolism, to facilitate the combination with photovoltaics efficiency in an electro-photo synthesis. Second, hydrogen-driven electrosynthesis is one of the most successful bioengineering energy generation formats. It bears outstanding features including high efficient bioenergy storage of up to 80% of electrical energy, long distance transportability with minimum energy loss, hydrogen oxidation in microbes linking to NAD^+^ reduction minimizing potential mismatch, and affordability due to lower cell-protein requirements of hydrogen oxidation. Third, electron transfer can extracellularly mediate electro-synthesis effectively. If this is made reproducible, for example, through a nanostructured surface to facilitate biofilm formation, it can skip the requirement of extended surface area and enhance hydrogen electron transfer.

Applied biological energy generation will be greatly enhanced by the innovation of multiple other technologies. To mention a few, novel technologies such as gene engineering, whole genome engineering, protein engineering, and biosensing will promote biofuel generation and development remarkably. As one of the most prominent applications of applied biology in sustainable energy, biofuel is expected to become the most deployed methodologies in solar energy capturing and storage with the lowest costs. Current challenges in biofuel development are the energy production efficiency and scale, capability exploration in cell self-assembly and replication control, and adverse environmental impacts. In the near future, biofuel is expected to improve and prolong traditional energy sources, to recycle and reproduce energy generating materials, and to enhance hybrid energy photosynthesis.

Commercialization of sustainable technologies in the agri-food market is ongoing, with several main focuses; biosensor commercialization, sensing technology commercialization, and intelligent food packaging commercialization. In biosensor commercialization, key factors determining its commercialization are simpler sample pretreatment, bioreceptor stability, multi-detecting properties, miniaturization, shorter testing time, wireless availability, and affordability [152]. Current commercially available biosensors for food safety and quality control, their vendors, and their targeted analytes are listed in Table 4. Common features of commercialized biosensors are their simple construction, smaller sizes, and ideal qualities for POC applications. Those commercialized food biosensors target food composition, process control, and food security including pathogens, allergens, toxins, contaminants, and additives [153]. For example, food quality biosensors target mainly the following metabolites: glucose, sucrose, glycerol, cholesterol, creatinine, alcohol, methanol, lactate, lactose, glutamate, malate, and ascorbic acid.

Compared to previous and current studied biosensors in academic laboratories, the biosensors under commercialization are far more less indicatory of the low success rates in agri-food-related biosensor development [152]. Limitations hindering biosensor development in food sector are considerable obstacles, such as issues in mass production, sensor lifetime, component integration, and handling practicability. The reasons behind these limitations are that most technologies applied in current and future food biosensing technology including nanotechnology, food material science, biomimetic chemistry, and microengineering, are in their infancy stages. A fundamental factor that determines the future of a biosensor is its safety in human health, which means that only those biosensors and related technologies with minimum or no human health impact will have their commercialization future in the coming years.

Commercialization of intelligent food packaging indicates urgent needs in novel and efficient methods to ensure food quality and safety, to economize packaging process, and to reduce food loss [97]. The most recent commercialized products in intelligent packaging include sensing indicators and sensor-enabled RFID tags. Sensing indicators are used to provide immediate information about the qualitative, semi-quantitative, and visual status of packaged foods through detecting increased color intensity, color change, or a dye diffusion level. These indicators can be categorized into gas (water vapor, ethanol, O_2_, and CO_2_), freshness, time-temperature, and thermochromic (color change based on temperature) indicators. Among them, thermochromic indicators are various specialized dynamic inks, such as touch activated thermochromic inks, cold activated thermochromic inks, and high temperature thermochromic inks, which are widely applied to intelligent packaging to ensure and alert consumers about the food status and qualities.

RFID in intelligent food packaging belongs to the category of automatic identification (auto ID) because its roles are similar to barcodes, magnetic inks, biometrics, and voice recognition, which is particularly useful in food supply chains and larger production networks [97]. An RFID tag is a device with a microchip and an antenna that carries product data. The potency of RFID is enlarged by biosensor technology because the carried product data can be communicated with biosensors. Three types of RFID tags are in use, including passive, semi-passive, and active tags, with differences in their power sources and data transfer distances. Giant supply chains from the U. S. market such as Walmart and BestBuy are promoting the application and development of RFID tags.

Sensor-enabled RFID tags bear the most promising future in these tag applications because they are faster, secure, productive, more efficient, and have better consumer preference [97]. They measure food-related properties such as temperature, pressure, pH, relative humidity, volatile compounds, light exposure, and gas concentrations; and they are practically useful for perishable foods like vegetables, fruit, fish, and meats. Sensor-enabled RFID tags have been commercialized to enable enhanced inventory and stock control, better cold-chain management, food loss reduction, better customer service, lower costs, and increased branding and profits in food industry. The main challenges are the application of multiple biosensors with various sensing properties into the same RFID tag, and the integration of sensor-enabled RFID tags into food packaging. The first prototype of RFID tags with biosensor integration and semi-passive flexibility has been developed in Europe in 2007; other RFID tags with biosensor integration to detect multiple food properties are currently under intensive development [154,155].

## 10. Conclusions

Within sustainability of food production, five top challenges are summarized: production challenge about food safety and security, quality challenge in food diversity and qualities, economic challenge in governing food system including its packaging and supply chain, environmental challenge including food waste processing, and engineering challenge in novel food creation and generation. This review focuses on biosensors and biosensing technologies with its applications in tackling all five top challenges. Biosensors with electrochemical impedance spectroscopy have been widely applied to sustainable food production. Other novel biosensors focus on powerful tunable features that are switchable between on/off status when responding to an external signal. The combination of electrochemical microfluidic and cell culture technologies represents a novel analytic technique in food analysis. Nanotechnology and its development in agriculture has been significantly expanded to various fields. These fields include food production, crop protection, pathogen and toxin detection, water purification, food packaging, wastewater treatment, and environmental remediation. POC technologies focus on rapid, simple, accurate, portable, and low-cost analytical instruments; and up-to-date concepts in POC technologies and multiple POC device candidates have been developed to meet these conceptual requirements.

Food safety and security are related to all steps in the food chains from food farming, production, process, packaging, transportation, and all the way to consumption. Biosensing technologies applied to the field of food safety and security have been developed for nutrient and qualities detection, pathogens detection, and toxin detection. In food packaging, the most advanced technologies are nanomaterials with their biosensing and antimicrobial properties, and intelligent and robotic technologies with their highly potency in automatic food packaging. Food supply chains are expected to provide adequate information to consumers and other concerned bodies about food attributes, animal welfare, GM issues, and country of origin. In this regard, food traceability systems are ideal in fulfilling the requirements because of the current issues existing in food traceability.

The generation of food waste is on its rise due to a growing global population, leading to increased food production and consumption. Among current food waste technologies, nanomaterials, bioactive compounds, biofuels, biodegradable plastics, and enzymes are the main points of focus. In current food production, contamination sources exist in agriculture chemical usage and other underestimated origins, which calls for the urgent development of biosensing instruments to assess and control food qualities and to reduce potential hazards. A combination of various technologies is required in food quality evaluation, such as combining microbial biosensors, DNA biosensors, biochemical DNA repair, and metabolic activation of toxin-like carcinogens.

Food engineering is a multi-disciplinary field combining physical sciences and product properties to generate processes and equipment, which converts raw agricultural materials and ingredients to convenient, safe, and nutritious food products. However, bioengineered nanomaterials and their application in food production is in its infancy with a pending issue because of its unknown properties on human health. Opportunities and challenges currently co-exist in food engineering as it sheds light on 21st century expectation and requirements. Major challenges in food engineering have been identified as its open innovation, virtualization, modeling, and social responsibility.

To summarize, major challenges from food sustainability focus on three fields: nanomaterials and their application challenge in sustainable agriculture, energy sustainability challenges, and the commercialization challenge of sustainable technology. A fundamental factor that determines the future of a biosensor is its safety in human health, which means that only those biosensors and related technologies with minimum or no human health impact will have commercialization futures. When developing a biosensor in food production sustainability, it is important to consider the urgent needs in ensuring food quality and safety, but it is also critically significant to ensure that the biosensor itself is safe for human health, because otherwise it has no future in its commercialization.

## Figures and Tables

**Table 1:** Estimated post-harvest handling and storage losses in percentage [54].

Region	Cereals	Roots & Tubers	Fruits & Vegetables
Europe	4	9	5
North America & Oceania	2	10	4
Industrialized Asia	10	7	8
Sub-Saharan Africa	8	18	9
North Africa, West & Central Asia	8	10	10
South, Southeast Asia	7	19	9
Latin America	4	14	10

Source: FAO 2011. Global food losses and food waste: extent, causes and prevention. Rome: Food and Agriculture Organization.

**Table 2:** Temperature and water activity requirements for fungal growth [54].

Species	Temperature (°C)			Water Activity (Aw)	
	Minimum	Optimum	Maximum	Minimum	Optimum
*Aspergillus ruber*	5	24	38	0.72	0.93
*A. amstelodami*	10	30	42	0.70	0.94
*A. flavus*	12	35	45	0.80	0.99
*A. fuminatus*	12	40	52	0.83	0.99
*A. niger*	10	35	45	0.77	0.99
*Penicillium martensii*	5	24	32	0.90	0.99

**Table 3:** Conditions for numbers of bacteria grown in milk [54].

Temperature (°C)	24 h	48 h	96 h	168 h
0	2400	2100	1850	1400
4	2500	3600	218,000	4,200,000
8	3100	12,000	1,480,000	
10	11,600	540,000		
15	180,000	28,000,000		
30	1,400,000,000			

**Table 4:** Commercially available biosensors for food quality and safety.

Target	Company	Country
Ethanol, Methanol, Glucose, Lactate, Glycerol	Analox Instruments	UK, USA
Water soluble vitamins, Veterinary residues (Chemical), and Mycotoxins	Biacore AB	Sweden
Microorganisms	Biomerieux	France
Microorganisms, Biochemical oxygen demand	Biosensores S.L.	Spain
Alcohol, Allergen, Acids, Sulfites	Biosentec	France
Allergens, Vitamins, Microorganisms	Biotech-IgG	Sweden
Microorganisms, Drug residue	Eurofins	Luxembourg
Glucose, Lactate, Ammonia, Pyruvate	Gwent Sensors	UK
Bio Profile chemistry analyzer	Nova Biomedical	USA
Ethanol, Malate, d-Lactate, l-Lactate, Glucose, Fructose	Tectronik	Italy
Glucose, l-Glutamate, l-Glutamine	Trace Analytics	Germany
Glucose, Sucrose, Ethanol, Lactose, l-lactate, Galactose, l-glutamate, H_2_O_2_, Glutamine, Choline	Yellow Springs Instruments	USA
